# Painting observation changes balance in patients with bilateral vestibulopathy

**DOI:** 10.1371/journal.pone.0336800

**Published:** 2025-12-05

**Authors:** Adéla Kola, Gaëlle Quarck, Olga Kuldavletova, Thomas Stoffregen, Pierre Denise, Antoine Langeard

**Affiliations:** 1 Université de Caen Normandie, INSERM, CYCERON, CHU de Caen, Normandie Université, COMETE U1075, Caen, France; 2 School of Kinesiology, University of Minnesota, Minneapolis, Minnesota, United States of America; University of Education, PAKISTAN

## Abstract

**Background:**

Bilateral vestibulopathy (BVP) is a disorder characterized by significant impairments in vestibular function, leading to changes in the kinematics of standing body sway. While previous studies have demonstrated that observing paintings can influence postural control in healthy adults, the effects of such visual stimuli on individuals with BVP remain unexplored. This study aimed to investigate the impact of observing an artistic painting on postural balance.

**Methods:**

Posture was assessed in 34 patients with bilateral vestibuloapthy compared to 30 healthy controls using three static balance conditions: (1) eyes open by fixating a cross, (2) eyes closed, and (3) while viewing an impressionist painting (Le Bassin aux nymphéas, harmonie verte, Monet, 1899). This painting presents a specific interest due to its perspective cues, providing depth information and spatial reference points, its absence of motion and salient focal point.

**Results:**

Patients with bilateral vestibulopathy, compared to healthy controls, had higher center of pressure standard deviation and amplitude in the anteroposterior (AP) direction eyes open and eyes closed but no differences between groups were detected when viewing the painting. In addition, the variation of the AP position of the center of pressure was reduced by 20.94% for standard deviation when the BVP patients were looking at the painting in comparison to the standard eyes open condition.

**Conclusions:**

These findings suggest that art observation can influence postural control in vestibular-defective patients. Further research will be needed to understand the basis of this effect and its possible relevance for rehabilitative treatment.

## Introduction

Vestibular disorders are associated with changes in postural activity. Bilateral vestibulopathy (BVP) is a rare pathology affecting approximately 28 cases per 100,000 individuals in 2008 in the United States [1]. This rare disorder is often idiopathic and, in some cases, may be related to ototoxicity induced by pharmacological agents such as gentamicin [2]. This disorder leads to increased body sway [1]. For patients with BVP, sway becomes more pronounced during stance on irregular or soft surfaces, and in low-light conditions [2]. Similarly, eye closure is associated with significant increases in sway in both the mediolateral (ML) and anterioposterior (AP) axes [3], highlighting the role of vision in postural control for these patients. However, the existing literature provides little insight into the control of standing posture among BVP patients in relation to naturalistic variations in visual activity.

Some studies have revealed that postural control is modulated according to the demands of visual tasks. For example, compared with looking a blank environment, reading a text reduces the magnitude of sway [4,5]. This effect persists in healthy elderly individuals [6,7], and in some clinical populations [8,9]. By contrast, adults with dementia do not modulate their sway in response to variations in visual tasks [10]. Similar effects of visual tasks on postural activity have been observed in relation to tasks that were based on visual graphics, rather than text [8]. In the present study, we investigated whether patients with BVP would modulate their standing body sway in relation to viewing visual graphics in the form of artistic paintings.

Artistic paintings, which evoke psychological states [11] and neural responses [12], hold promise for improving our understanding of the complex interaction between vision and postural balance in patients with BVP. In healthy individuals, it has been shown that the eye movements in front of Op Art may induce larger body sway [13], illustrating how abstract art can act through oculomotor and perceptual mechanisms. By contrast, figurative paintings seem to influence posture mainly through recognition processes and attentional engagement with representational content [14]. Also, emotion has an effect on postural control, and even more when the emotional valence is high [15]. Because figurative artworks often convey stronger emotional or narrative content than abstract ones, they may therefore have a higher impact on postural control. Thus, the kinematics of standing body sway can be influenced by viewing both figurative [16] and abstract art [17,18], although in the case of figurative art, the pathways involved are likely more numerous rather than fundamentally different.

We asked whether effects that have been observed in healthy populations would also be found in patients with BVP. Specifically, we examined the effects of looking at an impressionist painting on postural activity in individuals with BVP compared to healthy controls in comparison with standard postural assessment. By comparing postural control under different visual conditions (eyes open, eyes closed, and while viewing a painting), we investigated whether the postural responses of patients with BVP would be similar to those of healthy controls.

## Methods

### Participants

The study included 34 patients diagnosed with BVP and 30 healthy control participants (see Table 1). Ethical approval was obtained from the French Ethical Committee (Comité de Protection des Personnes de la Région Ouest I, ID-RCB 2022-AO1513-40). Patients were recruited from the *Association Française de Vestibulopathie Bilatérale* (www.afvbi.info). The BVP diagnosis followed the Bárány Society criteria, as described by Strupp et al. (2017) [1]. Disease duration was known for 18 patients, and was 25.72 ± 26.26 years on average. Available vestibulo-ocular reflex gain (n = 20) was at 0.326 ± 0.196 for the right canals and 0.323 ± 0.170 for the left canals. Controls subjects were matched according to sex, age and level of education. Only four BVP participants remained unmatched because no suitable controls were available within the study period. All participants were tested in the COMETE Laboratory at the University of Caen from 3 October 2022–18 January 2024 and provided written informed consent. All assessments were supervised by experts in posture-related research, with formal training and extensive experience in the use of this equipment [19–21].

**Table 1:** Subject’s characteristics (mean ± sd).

Characteristics	BVP	Controls
Sex	19 ♀; 15 ♂	17 ♀; 13 ♂
Age (years)	60 ± 13	59 ± 13
Level of education (years)	14.67 ± 2.51	14.83 ± 2.13
Height (cm)	169.6 ± 8.42	168.3 ± 9.16
Foot length (mm)	250.73 ± 16.59	252.90 ± 16.54

Level of education was assessed on a scale ranging from 0 (no primary education) to 17 (master’s degree and higher).

### Experimental protocol

Postural control was assessed using the Synapsys Posturography System® (SPS; Synapsys, Marseille, France; sampling frequency of 100 Hz). Safety rails were installed around the platform to ensure participants’ safety and to prevent falls. Participants stood barefoot on a force platform, with their feet positioned at a 30° angle [22], and were instructed to keep their arms relaxed at their sides without lifting their feet.

Participants performed 60-second tasks in three different visual conditions: 1) eyes open (EO), where participants fixated a cross positioned at eye level in front of them; 2) painting exploration, where participants visually explored an impressionist painting (*Le Bassin aux nymphéas, harmonie verte, Monet, 1899*), chosen for its depth cues, its impressionist art movement and its low motion content; and 3) eyes closed (EC), were participants stood with their eyes closed. Unlike the eyes open condition, in which participants fixated on a cross, the painting exploration condition involved no explicit focal point. In addition, the emotional and aesthetic qualities typically evoked by impressionist artworks might further modulate postural control, as emotional engagement has been shown to influence body sway. The tests were performed in a random order. The distance between the subject and the image (size: 172x113.5 cm) was 190 centimeters. We excluded from analysis trials on which the participant had any physical contact with the safety rails.

The SPS system calculated measures of postural balance, including amplitudes (ranges of maximum-minimum of the center of pressure (CoP) displacement) (CoP amplitude), and standard deviations (CoP SD) in the mediolateral (ML) and anteroposterior (AP) directions. Velocities (CoP velocity) in the ML and AP directions were calculated from the raw CoP positions.

### Statistical analyses

Robust linear mixed models were used to compare the group and task differences, controlling for sex, age, level of education, height, and foot length. When a Group × Condition interaction was significant, the Tukey method was used for multiple comparison corrections in post hoc analyses. The significance threshold was set at a p-value of 0.05. The Group × Condition were calculated with the painting condition as a reference. The statistical analyses were conducted in the R Studio Software (2023.12.1 + 402), using the rlmm package. Post-hoc comparisons were performed with marginal means and Cohen’s d was calculated using the residual standard deviation from the robust model (R package effectsize).

## Results

The data (S1 File) and results are summarized in Table 2 and in Supplementary Table (S1 Table). The terms Group (BVP patients vs. controls), Condition (eyes closed vs painting, eyes open vs painting), and the Group × Condition interaction were analyzed on the dependent variables (standard deviation, amplitude, and velocity in the AP and ML planes), while controlling for covariates including age, sex, education level, height, and foot length.

**Table 2:** Marginal means (±SE) by groups (controls and BVP patients) and conditions (painting, EO, EC).

		Painting			EO			EC		
		Controls (n = 30)	BVP (n = 32)	p	Controls (n = 30)	BVP (n = 34)	p	Controls (n = 30)	BVP (n = 29)	p
CoP SD (mm)	AP	4.67 ± 0.45	5.55 ± 0.42	.723	5.23 ± 0.45	7.02 ± 0.42	.045*	5.54 ± 0.45	7.67 ± 0.43	.009**
	ML	4.08 ± 0.39	4.80 ± 0.37	.774	3.87 ± 0.39	5.03 ± 0.37	.274	4.34 ± 0.39	5.78 ± 0.38	.097
CoP Amplitude (mm)	AP	25.03 ± 2.55	32.03 ± 2.41	.356	26.37 ± 2.55	37.31 ± 2.38	.024*	31.97 ± 2.55	45.30 ± 2.47	.003**
	ML	23.01 ± 2.40	27.06 ± 2.27	.830	21.81 ± 2.40	29.16 ± 2.25	.233	25.37 ± 2.40	36.08 ± 2.31	.018*
CoP Velocity (mm/s)	AP	8.38 ± 0.76	10.05 ± 0.72	.611	7.90 ± 0.76	10.68 ± 0.71	.090	12.68 ± 0.76	18.21 ± 0.74	<.001***
	ML	7.33 ± 0.74	8.26 ± 0.70	.945	6.91 ± 0.74	9.46 ± 0.69	.126	9.15 ± 0.74	11.55 ± 0.73	.195

BVP: bilateral vestibulopathy; EO: eyes open; EC: eyes closed; CoP: center of pressure; ML: mediolateral; AP: anteroposterior; SD: standard deviation; *: p < 0.05, **: p < 0.01, ***: p < 0.001.

### Anteroposterior evaluation

**Fig 1:**
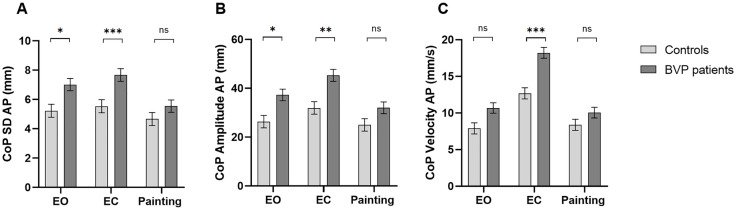
Anteroposterior CoP parameters of BVP and controls across three conditions (EO, EC, Painting). (A) CoP standard deviation; (B) CoP amplitude; (C) CoP velocity. BVP: bilateral vestibulopathy; CoP: center of pressure; AP: anteroposterior; SD: standard deviation; EO: eyes open; EC: eyes closed; ns: not significant. Values represent marginal means ± standard error (SE), controlling for sex, foot length, height, and level of education. Significant group differences were observed for CoP standard deviation in EO (p < .05*) and EC (p < .01**) conditions, CoP amplitude in EO (p < .05*) and EC (p < .01**) conditions, and CoP velocity for EC condition (p < .001***).

For CoP SD AP (Fig 1A), the main effect of Condition was significant (Painting vs. EC), β = 0.87, t = 2.39, p = .018. CoP SD AP was greater in EC compared to the Painting condition. The Group × Condition (Painting vs. EC) interaction was significant, β = 1.26, t = 2.43, p = .016. Post-hoc tests revealed that, in patients, CoP SD AP was greater in the EO compared to the Painting condition (p < .001, Cohen’s d = 1.074). Moreover, patients had greater CoP SD AP than the Control group in EO (p = .045, Cohen’s d = 1.307) and EC conditions (p = .009, Cohen’s d = 1.556) but not in the Painting condition. The main effect of Group was not significant.

For CoP Amplitude AP (Fig 1B), the main effect of Condition was significant (Painting vs. EC), β = 6.93, t = 3.55, p = .001. CoP Amplitude AP was higher in EC compared to the Painting condition. Patients had higher CoP Amplitude AP than the Control group. A Group × Condition (Painting vs. EC) interaction was significant, β = 6.34, t = 2.29, p = .024. Post-hoc tests indicated that CoP Amplitude AP was higher in EC compared to in EO and Painting conditions for both patients (p < .001, Cohen’s d = 1.086 and p < .001, Cohen’s d = 1.802 respectively) and the Control group (p = .048, Cohen’s d = 0.760 and p = .005, Cohen’s d = 0.941 respectively). Moreover, patients had higher CoP Amplitude AP in both EO (p = .024, Cohen’s d = 1.485) and EC (p = .003, Cohen’s d = 1.811) conditions compared to the control group, but not in the Painting condition. The main effect of Group was not significant.

For CoP Velocity AP (Fig 1C), the main effect of Condition was significant (Painting vs. EC), β = 4.29, t = 7.07, p < .001. AP-CoP-velocity was greater in EC compared to the Painting condition. The Group × Condition (Painting vs. EC) interaction was significant, β = 3.86, t = 4.46, p < .001. Post-hoc tests showed that, in the Control group, CoP Velocity AP was higher in EC compared to in EO (p < .001, Cohen’s d = 2.079) and the Painting condition (p < .001, Cohen’s d = 1.871). For patients, CoP Velocity AP was higher in EC compared to the Painting condition (p < .001, Cohen’s d = 3.561). Moreover, patients had greater CoP Velocity AP than the Control group, only in EC condition (p < .001, Cohen’s d = 2.411). The main effect of Group was not significant.

### Mediolateral evaluation

**Fig 2:**
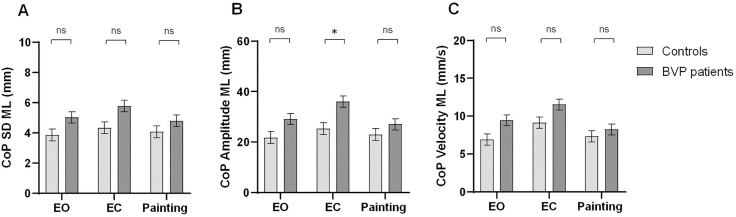
Mediolateral CoP parameters of BVP and controls across three conditions (EO, EC, Painting). (A) CoP standard deviation; (B) CoP amplitude; (C) CoP velocity. BVP: bilateral vestibulopathy; CoP: center of pressure; ML: mediolateral; SD: standard deviation; EO: eyes open; EC: eyes closed; ns: not significant. Values represent marginal means ± standard error (SE), controlling for sex, foot length, height, and level of education. Significant group differences were observed for CoP amplitude in EC condition (p < .05*).

For CoP SD ML (Fig 2A), no significant effects of Condition, Group, or Group × Condition interaction were found.

For CoP Amplitude ML (Fig 2B), a Group × Condition (Painting vs. EC) interaction was significant, β = 6.65, t = 2.80, p = .006. Post-hoc tests showed that patients had a greater CoP Amplitude ML than the Control group in EC condition (p = .018, Cohen’s d = 1.695). Also, patients had greater CoP Amplitude ML in the EC condition compared to the Painting (p < .001, Cohen’s d = 1.427) and EO conditions (p < .001, Cohen’s d = 1.095). The main effect of Group and the main effect of Condition were not significant.

For CoP Velocity ML (Fig 2C), the main effect of Condition was significant (Painting vs. EC), β = 1.82, t = 2.59, p = .011. CoP Velocity ML was greater with the eyes closed than when viewing the Painting. The main effect of Group and the Group × Condition interaction were not significant.

## Discussion

This study aimed to examine the effects of viewing a painting on postural activity in individuals with BVP compared to healthy controls. We examined body sway while participants stood with eyes closed (EC), with eyes open while viewing a fixation cross (EO), and eyes open while viewing a painting (Painting). Our results replicated the finding that BVP is associated with altered postural activity in both the mediolateral and anterior-posterior axes [3]. Our results also replicated previous studies that have demonstrated an effect of artistic paintings on standing posture [16,17]. Finally, we found that postural responses to viewing artistic paintings differed between BVP patients and healthy controls.

### General effects of BVP

Previous research has shown that standing body sway in patients with BVP differs from healthy controls. When standing on a flat stationary surface, Baloh et al. [3] reported that BVP patients exhibited greater amplitude and velocity of sway (compared to healthy controls). In the mediolateral axis, this effect was observed only when the eyes were open, while in the anterior-posterior axis, the effect was observed both with eyes open and with eyes closed. In the present study, a similar effect was observed only for sway amplitude in the mediolateral axis, and only when the eyes were closed. By contrast, in the anteroposterior axis, we found a significant main effect of groups for sway amplitude. In other words, sway amplitude was greater for BVP patients, both when the eyes were closed and when they were open.

### Effects of artistic paintings on healthy controls

Cox and Klaveren [17,18] examined the spatial dynamics of sway by analyzing the mean position of the CoP (i.e., lean) and its positional variability. Their studies compared sway during the viewing of paintings by two 20^th^ century artists, but did not include a control condition without artwork viewing. As a result, their findings are not directly comparable to ours. Kapoula and Gaertner [16] assessed sway during the viewing of artistic paintings in comparison to a control condition where participants viewed a fixation cross. They found that the velocity of CoP displacements was significantly greater during viewing of 20^th^ century paintings than when viewing the fixation cross. In contrast, in our healthy control group, we did not observe any statistically significant differences in sway between viewing the painting and viewing the fixation cross. In this sense, our results do not replicate those reported by Kapoula and Gaertner. In their study, they used another Monet painting that depicted a woman positioned at the center, providing a salient focal point and a strong sense of motion through the suggestion of wind in her veil and dress, whereas our stimulus was a landscape painting without a central moving figure, evoking instead a sense of depth and perspective. In addition, the exposure time in our protocol (1 minute) was more than twice as long as in Kapoula and Gaertner’s study (25.6 s). These methodological differences may explain for the divergent results. This discrepancy may be related to differences in exposure time (60 s vs. 25.6 s) or to the nature of the paintings. Although both studies used paintings by Monet, theirs featured a standing figure with strong implied motion, while ours used a tranquil landscape with strong perspective cues. However, we found statistically significant differences in sway among healthy controls when comparing the viewing of the painting to standing with eyes closed. Nevertheless, these effects are not relevant to the question of whether artistic paintings influence postural control.

### Effects of artistic paintings on BVP patients

For BVP patients, we observed several statistically significant differences in sway between viewing the painting and standing with eyes closed (EC condition).

Notably, for BVP patients, the positional variability of the CoP in the AP axis was greater when viewing the fixation cross (EO condition) than when viewing a painting, for amplitude and standard deviation. This effect provides direct evidence on a differential influence of the artistic painting on postural control, and represents the main result of our study. It is particularly interesting to note that sway was influenced by viewing the painting (relative to the fixation cross) in BVP patients but not in healthy controls, especially in the AP direction by the reduced standard deviation of 20.94% when viewing the painting. This contrast suggests that BVP patients may exhibit sensitivity or reactivity to subtle variations in visual stimuli, in this case, the differences between a painting and a fixation cross. As mentioned, many studies have demonstrated that postural sway is systematically influenced by subtle variations in visual targets and visual tasks in healthy adults and children [7,23,24]. Our findings suggest that similar effects may also occur in BVP patients, and that these effects could be more pronounced for patients than for healthy controls. This observation highlights the need for further research on how variations in visual task influences postural control in BVP patients.

Also, paintings with clear depth cues, such as the one used in the present work, could provide visual anchors that could help BVP patients compensate for their vestibular loss and support postural control. Interestingly, some patients even reported a sensation of “being drawn” into the painting. This suggests that, despite their vestibular impairment, BVP patients preserve a sense of embodiment [25].

Furthermore, recent work has shown that embodiment can also be elicited by abstract art [17], reinforcing the idea of an embodied interaction between the viewer and the artwork. However, our study did not directly assess subjective feelings or bodily sensations experienced while viewing the painting. Future studies should therefore include specific questionnaires to evaluate both the bodily dimension of embodiment and the emotional experience associated with observing art, in order to better understand how these factors contribute to postural responses.

Finally, this change in postural balance in patients with BVP could be related to the way paintings guide visual attention and stabilise gaze. Patients with BVP typically have less stable gaze due to an impaired vestibulo-ocular reflex [1]. Viewing a painting, however, requires more precise dynamic control of the visual system than viewing a fixation cross, similar to studies in which sway was decreased during reading of text [4,5]. Visual examination of the painting is similar to text reading that it requires precise control of sequential changes in fixation. In text reading, a series of individual words is fixated, while in viewing art a series of individual features (e.g., lines, colors, faces, objects) is fixated. Precise changes in fixation are not required when viewing a fixation cross. Moreover, depth-rich paintings such as those by Monet offer perspective cues and visual flow that can serve as ‘anchor points’ for the gaze, encouraging structured visual exploration. By requiring stability of gaze, such stimuli may improve the functional integration of visual and postural control, that is, the ability of postural control to contribute to stable visual exploration. Future studies could test whether different Monet paintings, or other artistic styles, elicit specific effects.

These results have important clinical implications. They suggest that artistic paintings with clear depth cues may be used as visual stimuli that improve visual-postural integration in BVP patients, potentially complementing vestibular rehabilitation protocols and providing a more engaging alternative to conventional visual fixation tasks. From a research perspective, our findings highlight the need to further investigate how complex visual environments interact with postural control in vestibular disorders, including the roles of gaze stability, attentional focus, embodiment, and emotional engagement.

Moreover, these findings highlight the potential benefits of art observation on postural balance in BVP patients. Viewing art provides complex and dynamic sensory input, promoting a sense of embodiment and environment perception. Monet’s impressionist paintings, known for their depth cues, exemplify artwork that could support visual-postural integration in BVP patients, offering a novel, ecological approach to understand and manage balance in vestibular disorders. Emotional and aesthetic engagement elicited by such artworks may also contribute to postural regulation. By expanding our understanding of how art influences postural control, we could develop targeted interventions for improving balance and reducing fall risk in vulnerable populations.

Several limitations should be acknowledged, including the lack of eye-tracking and subjective measures of embodiment and emotion. Future studies incorporating these measures, as well as variations in painting style, exposure time, and visual motion cues, would allow for a more comprehensive understanding of the mechanisms through which artistic paintings influence balance in BVP patients.

## Conclusion

Overall, these findings suggest that viewing art may enhance the functional integration of postural control with visual exploration in BVP, offering a potential non-pharmacological intervention. While the absence of eye-tracking and subjective measures such as embodiment, aesthetic experience, or emotion limits the interpretation of these findings, future studies manipulating painting style, exposure duration, and visual motion cues will help clarify underlying mechanisms. Taken together, this research may open new perspectives for using art-based approaches to support balance in individuals with BVP.

## Supporting information

S1 File Raw data on postural parameters for patients with bilateral vestibulopathy and controls. (XLSX)

S1 Table Group, Condition and Group x Condition effects on postural parameters. β: regression coefficient; se = standard error; t: t-value; p: p-value; BVP: bilateral vestibulopathy; EO: eyes open; EC: eyes closed; CoP: center of pressure; ML: mediolateral; AP: anteroposterior; SD: standard deviation; *: p < 0.05; **: p < 0.01; ***: p < 0.001.
(DOCX)
