# Supplementary material for: Painting observation changes balance in patients with bilateral vestibulopathy
Source: PLoS One. 2025 Dec 5;20(12):e0336800. doi: 10.1371/journal.pone.0336800 (PMC12680332; doi:10.1371/journal.pone.0336800)
Supplement: S1 Table — β: regression coefficient; se = standard error; t: t-value; p: p-value; BVP: bilateral vestibulopathy; EO: eyes open; EC: eyes closed; CoP: center of pressure; ML: mediolateral; AP: anteroposterior; SD: standard deviation; *: p < 0.05; **: p < 0.01; ***: p < 0.001. (DOCX) [file pone.0336800.s002.docx]

| **S1 Table. Group, Condition and Group x Condition effects on postural parameters.** |  | Group effect (BVP *vs* controls) | | | | EO *vs* Painting | | | | EC *vs* Painting | | | | | Group*(EO *vs* Painting) | | | | Group*(EC *vs* Painting) | | | |
| --- | --- | --- | --- | --- | --- | --- | --- | --- | --- | --- | --- | --- | --- | --- | --- | --- | --- | --- | --- | --- | --- | --- |
|  |  | β | *se* | t | p | β | *se* | t | p | β | *se* | t | p | β | | *se* | t | p | β | *se* | t | p |
| CoP SD (mm) | AP | 0.88 | 0.62 | 1.41 | 0.162 | 0.56 | 0.36 | 1.53 | 0.127 | 0.87 | 0.36 | 2.39 | **0.018*** | 0.91 | | 0.50 | 1.82 | 0.072 | 1.26 | 0.52 | 2.43 | **0.016** |
|  | ML | 0.72 | 0.55 | 1.32 | 0.189 | -0.21 | 0.26 | -0.81 | 0.419 | 0.26 | 0.26 | 0.99 | 0.323 | 0.44 | | 0.36 | 1.20 | 0.232 | 0.71 | 0.37 | 1.91 | 0.058 |
| CoP Amplitude (mm) | AP | 6.99 | 3.54 | 1.97 | 0.051 | 1.34 | 1.95 | 0.69 | 0.493 | 6.93 | 1.95 | 3.55 | **<0.001**** | 3.93 | | 2.71 | 1.45 | 0.148 | 6.34 | 2.77 | 2.29 | **0.024*** |
|  | ML | 4.05 | 3.34 | 1.21 | 0.226 | -1.19 | 1.67 | -0.71 | 0.477 | 2.36 | 1.67 | 1.41 | 0.160 | 3.29 | | 2.32 | 1.42 | 0.159 | 6.65 | 2.38 | 2.80 | **0.006**** |
| CoP Velocity (mm/s) | AP | 1.68 | 1.06 | 1.58 | 0.161 | -0.48 | 0.61 | -0.78 | 0.434 | 4.29 | 0.61 | 7.07 | **<0.001***** | 1.10 | | 0.84 | 1.30 | 0.195 | 3.86 | 0.86 | 4.46 | **<0.001***** |
|  | ML | 0.93 | 1.02 | 0.91 | 0.366 | -0.42 | 0.70 | -0.60 | 0.551 | 1.82 | 0.70 | 2.59 | **0.011*** | 1.62 | | 0.97 | 1.66 | 0.100 | 1.47 | 1.00 | 1.47 | 0.144 |

*β: regression coefficient; se= standard error; t: t-value; p: p-value; BVP: bilateral vestibulopathy; EO: eyes open; EC: eyes closed; CoP: center of pressure; ML: mediolateral; AP: anteroposterior; SD: standard deviation ; * : p<0.05; ** : p<0.01; *** : p<0.001*
